# *In vivo* Measurement of Intraosseous Vascular Haemodynamic Markers in Human Bone Tissue Utilising Near Infrared Spectroscopy

**DOI:** 10.3389/fphys.2021.738239

**Published:** 2021-09-24

**Authors:** Robert Meertens, Karen M. Knapp, William David Strain, Francesco Casanova, Susan Ball, Jon Fulford, Clare Thorn

**Affiliations:** ^1^College of Medicine and Health, University of Exeter, Exeter, United Kingdom; ^2^NIHR Applied Research Collaboration South West Peninsula (PenARC), College of Medicine and Health, University of Exeter, Exeter, United Kingdom; ^3^NIHR Exeter Clinical Research Facility, College of Medicine and Health, University of Exeter, Exeter, United Kingdom

**Keywords:** near infrared spectroscopy, bone, tibia, haemodynamic analysis, vascular physiology

## Abstract

**Objective:** Poor vascular health is associated with reduced bone strength and increased risk of fragility fracture. However, direct measurement of intraosseous vascular health is difficult due to the density and mineral content of bone. We investigated the feasibility of using a commercially available continuous wave near infrared spectroscopy (NIRS) system for the investigation of vascular haemodynamics in human bone *in vivo*.

**Approach:** An arterial occlusion (AO) protocol was developed for obtaining haemodynamic measurements of the proximal tibia and lateral calf, including assessment of the protocol’s intra operator reproducibility. For 36 participants, intraosseous haemodynamics derived by NIRS were compared to alternative tests of bone health based on dual x-ray absorptiometry (DXA) testing and MRI.

**Main Results:** Near infrared spectroscopy markers of haemodynamics of the proximal tibia demonstrated acceptable reproducibility, comparable with reproducibility assessments of alternative modalities measuring intraosseous haemodynamics, and the use of NIRS for measuring muscle. Novel associations have been demonstrated between haemodynamic markers of bone measured with NIRS and body composition and bone mineral density (BMD) measurements obtained with both DXA and MRI.

**Significance:** Near infrared spectroscopy provides inexpensive, non-invasive, safe, and real time data on changes in oxygenated and deoxygenated haemoglobin concentration in bone at the proximal tibia. This study has demonstrated the potential for NIRS to contribute to research investigating the pathophysiological role of vascular dysfunction within bone tissue, but also the limitations and need for further development of NIRS technology.

## Introduction

Bone is a dynamic and vascular tissue type that is constantly self-regulating with around 5% of the body’s blood volume supplying the skeleton (Prisby, 2017). The vascular supply is integral to the regulation of bone formation and resorption. Mechanisms which control angiogenesis within bone (triggers such as cytokines and growth factors) also activate bone regulation metabolism (Prisby, 2019). Likewise, arterioles lie at the centre of bone resorption bays and play the primary role in mediating resorption prior to reformative osteoblastic activity (Laroche, 2002).

Epidemiological links are established between arteriosclerotic diseases, reduced bone mineral density (BMD) and fracture incidence. Osteoporotic bone has been demonstrated to have increased fatty involution, loss of arterioles, and reduced capillary density at remodelling sites (Laroche, 2002; Kristensen et al., 2013). Accelerated bone resorption in post-menopausal females has established links to oestrogen reduction affecting both resorption rates and microvascular health (Dubey et al., 2005). Murine studies involving oophorectomy have demonstrated the reduction of oestrogen levels precipitating vascular dysfunction and reduced capillary density prior to changes to bone quality, suggesting a causal pathway (Roche et al., 2014; Wáng et al., 2015).

The intraosseous vascular supply of bone tissue *in vivo* is difficult to measure using existing image modalities due to bone’s density and high mineral content (6). Imaging protocols may involve nuclear medicine scans, positron emission tomography (PET), or MRI. Limitations of these tests include their expense, limited clinical access, and logistical safety considerations, involving invasive injections, and their inability to facilitate repeated measurements over time easily (Pifferi et al., 2004; Binzoni and Spinelli, 2015). Furthermore, the oxygenation status of bone cannot be measured with these techniques and therefore neither can markers of oxygen consumption metabolism (Binzoni et al., 2006).

**Figure 1:**
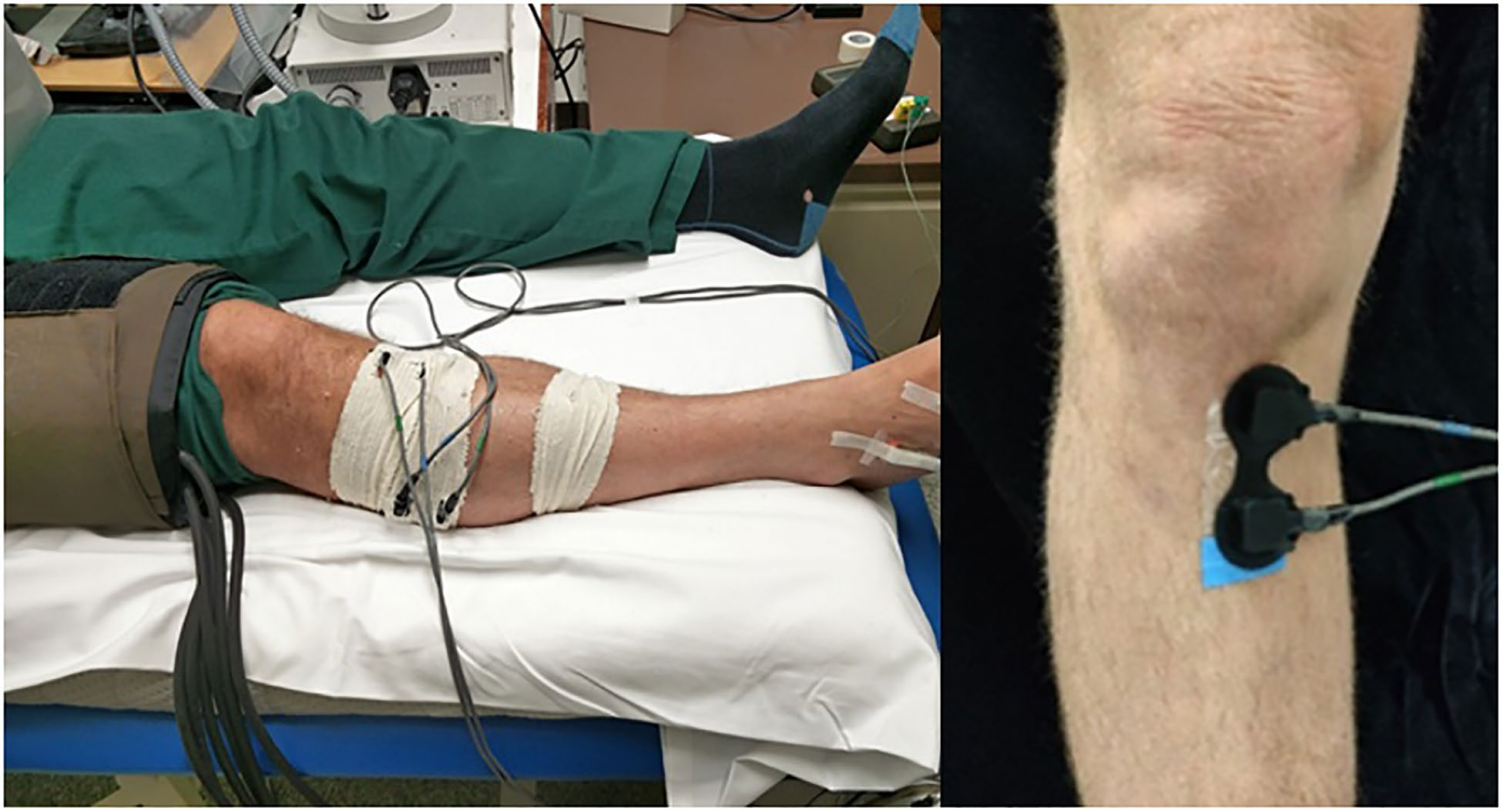
Arterial occlusion (AO) set up. Occlusion is set at the distal femur with NIRS optodes positioned at the right proximal tibia and lateral calf, secured and shielded with tubular bandages. A temperature optode and O_2_C optode is placed at the dorsal surface of the right foot. Inset: Example placement of NIRS optodes on the medial plane of the right proximal tibia. Measurements were taken at the left leg unless prohibited by a previous unilateral medical issue (such as previous fracture, varicose veins, etc.).

Near infrared spectroscopy (NIRS) is a potential technological solution, with a previous systematic review by the authors highlighting the breadth of possible applications (Meertens et al., 2018). NIRS involves transmitting and receiving near infrared light through the skin using non-invasive optodes at a specific anatomical site and takes advantage of differences in the absorption spectrum of oxygenated and deoxygenated haemoglobin in real time. The optodes provide the transmission of light at wavelengths of 735, 810, and 850nm from light emitting diodes (LED) into the tissue and the collection of backscattered light from the tissue using photodiodes for spectroscopic analysis (see Figure 1). This provides a measure of the tissue oxygen saturation as well as changes in haemoglobin volume indicative of vascular health in the tissue. This non-invasive technique enables the assessment of changes in tissue haemodynamics in response to different stimuli, such as a medical drug, simulated ischaemic occlusion, or exercise. NIRS has been shown to record data through bone to depths of up to 3cm (Aziz et al., 2010; Meertens et al., 2018).

If NIRS measurements can be demonstrated to represent valid and reliable markers of intraosseous haemodynamics, it would open the opportunity for new pathways in non-invasive skeletal research to directly investigate the potential effects of haemodynamic changes on bone health. This may help elucidate causal pathways to common and debilitating skeletal diseases such as osteoporosis, blood-born cancers, and osteoarthritis, furthering our understanding of skeletal disease and potential treatment options. NIRS could offer complimentary markers of intraosseous haemodynamics alongside existing metrics of bone health such as bone mineral density, analysis of bone microarchitecture, and metabolic markers of bone metabolism (Sordillo et al., 2015; Meertens et al., 2018).

A small body of evidence exists demonstrating NIRS can take measurements representative of superficial bone tissue, but with varied scientific methods that generally only compare results with soft tissue comparators (Meertens et al., 2018). The aim of this study was to assess an arterial occlusion (AO) protocol for acquiring markers of the intraosseous haemodynamic properties of the proximal tibia using a commercially available continuous wave NIR spectrometer. Within this assessment, we sought to gain confidence the tibia was truly represented by these markers, to examine their intra operator reliability, and to explore associations of these markers with NIRS results from the lateral calf muscle, measures of BMD and body composition [using dual x-ray absorptiometry (DXA)], and MRI scans of the measurement site.

## Materials and Methods

### Participants

Participants were recruited from the public with ethical approval (NHS Health Research Authority reference: 16/SW/0254). Written informed consent was obtained from all participants prior to inclusion in the study according to the Declaration of Helsinki. Inclusion criteria included being normotensive, non-smoking, Caucasian adults with a body mass index (BMI) of <35kg/m^2^. Exclusion criteria included contraindications to MRI; pregnancy; any history of recent serious injury or disease in the legs in the past 12months (including symptomatic deep vein thrombosis, or active claudication); active medical treatment for osteoporosis or any bone related health condition (such as bisphosphonates or hormone replacement therapy); and, recent use of glucocorticoids at a daily dose of 2.5mg or greater. Participants were screened for any known history of fracture, osteoporosis, metabolic bone disease, cardiovascular disease, current medications, and menopausal status (if female). To minimise natural physiological variation, participants were tested in the early morning and abstained from food and caffeinated drinks 2h prior to testing (Näslund et al., 2011; Thorn and Shore, 2016). Strenuous exercise was limited 48h prior to testing.

### Arterial Occlusion Testing Protocol

The AO protocol was considered most likely to be applied objectively and has the most precedence for *in vivo* intraosseous haemodynamic measurements (Binzoni et al., 2002, 2003; Klasing and Zange, 2003; Aziz et al., 2010; Farzam et al., 2014). A NIRO-200NX NIR spectrometer (Hamamatsu, Japan) was utilised allowing real time measures (at 1s intervals) of percentage tissue oxygenation saturation [tissue oxygenation index (TOI)] utilising a spatially resolved spectroscopy algorithm. The Modified Beer Lambert (MBL) law was used to calculate absolute change in oxygenated haemoglobin concentration (ΔO_2_Hb; measured in μM.cm); absolute change in deoxygenated haemoglobin concentration (ΔHHb; measured in μM.cm); and, relative change in total blood volume [normalised total haemoglobin index (nTHI); unitless; Elwell, 1995].

Initial physiological measurements taken included BMI, resting brachial blood pressure, ankle brachial index, and leg circumference at the knee, calf, and ankle, acquired using standardised protocols. Physiological monitoring during the AO protocol included digital heart rate and arterial oxygen saturation, and superficial temperature at the foot to rule out confounding systemic changes during the testing period, as described below.

The participant was positioned supine with the leg and foot in a relaxed neutral position (Figure 1). Measurements were obtained from the left leg unless the right leg was preferable (for example, due to the presence of varicose veins or previous fracture in the left leg). At the proximal tibia, the proximal NIRS optode was placed just medial to the palpable tibial tuberosity with both the transmitting and receiving optodes on the medial plane of the tibia, medial to the anterior tibial ridge. Comparative NIRS measures of the haemodynamics in muscle were also taken over the lateral head of the gastrocnemius muscle, an established site of NIRS measurement, to confirm any metabolic differences in bone and muscle tissue types. Interoptode spacing between the transmitting and receiving optodes of 40mm was used, providing an estimated 20mm depth of the sampled tissue volume (Aziz et al., 2010). NIRS optodes were shielded from ambient light with designated optode holders and tubular bandages. A fast inflating cuff was positioned at the distal femur to undertake a 4-min AO to the lower leg. Cutaneous TOI and blood flux measurements were also continuously monitored at the dorsal surface of the foot by white light spectroscopy and laser Doppler flowmetry provided by an O_2_C system (Oxygen to See; LEA Medizintechnik, Gießen, Germany) to confirm full arterial occlusion.

After a 15-min acclimatisation period in the supine position in a temperature controlled laboratory at 22.5+/−0.5°C, measurements taken were during a 2min baseline period before arterial cuff inflation, 4min during a 200mmHg AO, and 5min after cuff deflation. Successful AO execution was assessed based on mirrored change in O_2_Hb and HHb during the occlusion, attributable to oxygen metabolism within a closed vascular system. Acceptable AO also required a change of total haemoglobin concentration (nTHI) of less than 15% across the 4min AO period, suggesting stable blood volume during AO (Bopp et al., 2014). A successful AO was confirmed by reduction of skin blood flux to biological zero during occlusion (DO; Kernick et al., 1999). AO measurements were repeated on separate occasions within a 4-week period. All NIRS testing was performed by the same researcher, blinded to previous test results.

### Haemodynamic Markers

**Figure 2:**
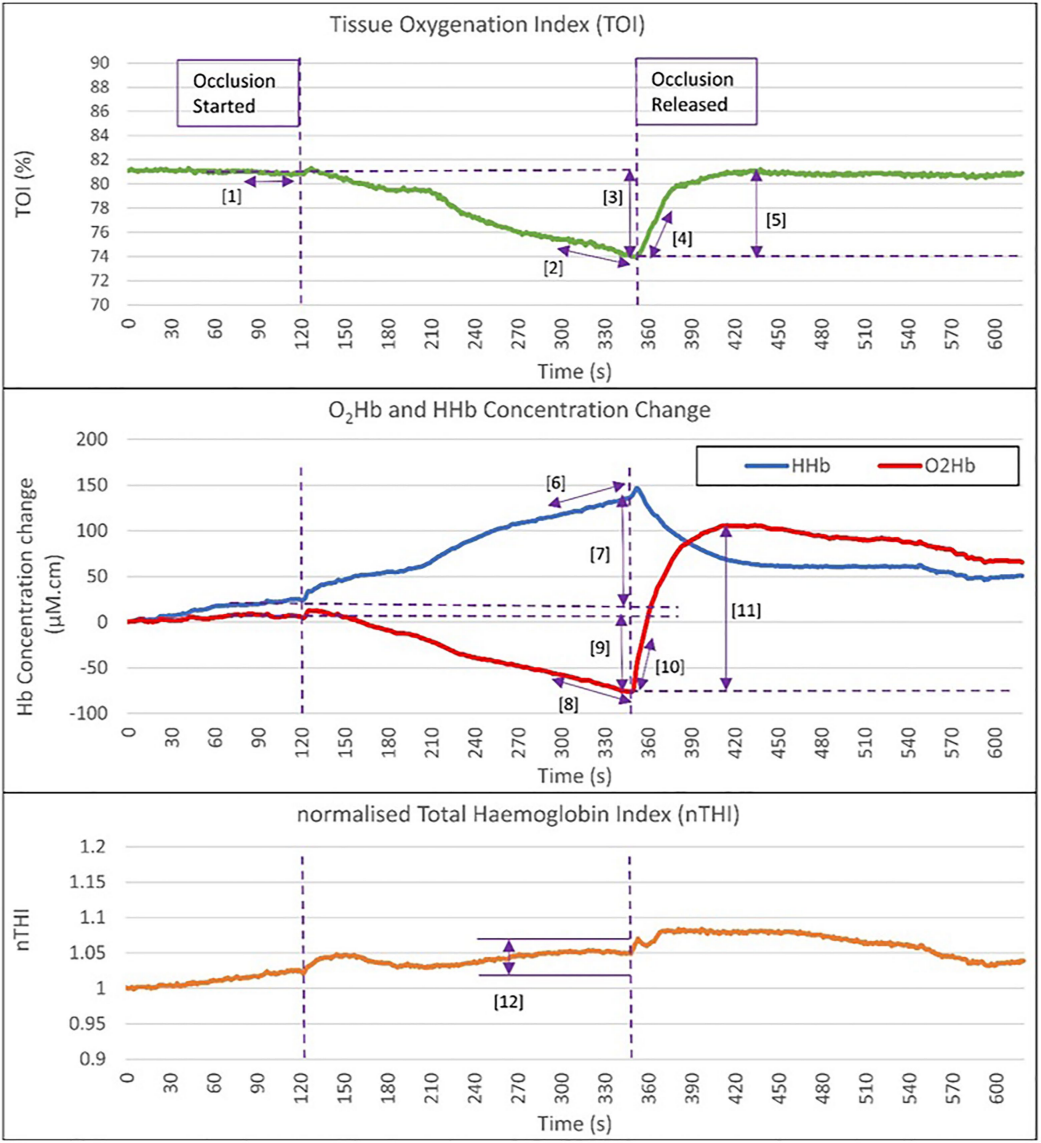
Graphical representation of the haemodynamic NIRS markers assessed during occlusion and post occlusion release. Haemodynamic markers are: (1) Baseline TOI at rest prior to the occlusion [TOI_rest; (%)]. (2) Rate of TOI decrease in the last 60s of occlusion [TOI_DO_ 60s (%/s)]. (3) Absolute change in TOI DO [TOI_DO_absΔ (%)]. (4) Rate of TOI increase in first 20s PO release [TOI_PO_20s (%/s)]. (5) Maximal absolute change in TOI post occlusion [TOI_PO_absΔ (%)]. (6) Rate of HHb increase in the last 60s of occlusion [HHb_DO_60s (μM.cm/s)]. (7) Absolute change in HHb during occlusion [HHb_DO_absΔ (μM.cm)]. (8) Rate of O_2_Hb decrease in the last 60s of occlusion [O_2_Hb_DO_60s (μM.cm/s)]. (9) Absolute change in O_2_Hb DO [O_2_Hb_DO_absΔ (μM.cm)]. (10) Rate of O_2_Hb increase in first 20s post occlusion release [O_2_Hb_PO_20s (μM.cm/s)]. (11) Maximal absolute change in O_2_Hb post occlusion [O_2_Hb_PO_absΔ (μM.cm)]. (12) Observation that the change in nTHI is less than 5% during the 60s period and within 15% change across the 4min occlusion. TOI, total oxygenation index; HHb, deoxygenated haemoglobin; O_2_Hb, oxygenated haemoglobin; DO, during occlusion; and PO, post occlusion.

Resting mean TOI measurements were averaged over a 20-s artefact free period immediately prior to execution of AO. Oxygen extraction DO was derived from the absolute change in TOI, O_2_Hb, and HHb over the 4 min AO, as well as linear rates of change during the final 60s of the occlusion. These rates of change were conditional on an nTHI change of less than 5% within the 60s period of measurement (in addition to the condition of less than 15% nTHI change across the 4min AO), ensuring blood volume changes did not confound these measurements. These data were also excluded if they were non-linear (i.e., Pearson’s *r*-value of <0.90). Post occlusion (PO) recovery was represented by markers derived from the initial linear rate of recovery (over 20s PO release) and the maximum extent of haemodynamic change PO release indicated by TOI and O_2_Hb parameters. Haemodynamic markers are presented graphically in Figure 2.

### Dual X-Ray Absorptiometry

Dual x-ray absorptiometry scans were performed using a GE Lunar Prodigy Advance 2005 DXA scanner, using Encore 11.40.004 software. Protocols involved whole body scans and dedicated scan protocols of the hip and lumbar spine as per accepted clinical positioning standards (Centers for Disease Control and Prevention, 2007). BMD measurements were reported in areal density (g/cm^2^) to allow comparison within the cohort of participants, as opposed to population based T-scores. From the whole body scans, measurements of areal density from the whole body, both legs combined, and the measured lower leg were taken. Whole body and lower leg composition was calculated, including percentage bone, lean tissue, and fat body mass.

### MRI

MRI scanning of the proximal tibia was performed using a Phillips Intera (1.5T) scanner with a dual element “Sense-Flex” coil. Sagittal and axial anatomical scans were performed using proton density protocols with fat suppression. Coronal scans were performed using a T1 spoiled gradient echo protocol with water selective excitation. A cod liver oil tablet was placed at a point marked on the skin of the proximal tibia, representing the site of the NIRS optodes from the previously obtained NIRS measurements. Total bone area and cortical bone were measured using freehand region of interest (ROI) placement from axial images as markers of cortical bone thickness and bone volume at the measurement site.

An MRI spectroscopy (MRS) protocol assessed relative fat content in marrow at the proximal tibia. A point resolved spectroscopy sequence (PRESS) was adopted with volume of interest 10×10×30mm within the bone marrow, repetition time of 2000ms, and echo time of 46ms with 96 acquisitions. Using the spectroscopic data obtained, a percentage fat fraction was calculated by dividing the integral of the fat peak (representing the area under the curve) by the sum of the water and fat peak integrals (Wáng et al., 2015; Karampinos et al., 2017).

### Data Analysis

Normality of data was checked using the Shapiro Wilks test. Reproducibility assessment included calculating root mean square SD (RMSSD) of within participant results, used to inform a “within participant” root mean square coefficient of variation (RMSCV; Bland, 2006). Intraclass correlation (ICC) was estimated using a one-way random effects model. Repeatability coefficients were calculated, which represent the maximum difference between repeat measurements that will only be exceeded in 5% of repeat measurements, assuming no physiological change in the participants bone health (Bartlett and Frost, 2008).

Pearson’s correlation assessed for association between tibial NIRS results and alternative bone health markers. A sample size of 36 participants was targeted to allow detection of a Pearson’s correlation co-efficient of 0.8 or higher with a 95% CI of 0.60–0.91 (Doros and Lew, 2010). Paired *t*-tests assessed for statistically significant differences between calf and tibial haemodynamics with a threshold *p*-value of 0.05 following confirmation of comparable variance using the Levene test. Comparison between sexes were performed using independent *t*-tests.

## Results

### Participant Data

Table 1 summarises the demographic data of all participants (*n*=41), with a separate summary of those who went on to undertake all testing of bone health (*n*=36), and sub groups of the latter based on sex. Only one participant had a history of recent low impact fracture. All participants reported no known history of metabolic bone disease, stroke or transient ischaemic attack, arrhythmias, cardiovascular disease, or renal disease. All 14 female participants were post-menopausal for a mean of 14.4years (range 6months to 35years). There was little evidence of demographic differences between sexes. Females were significantly shorter and with lower body weight, but with no significant difference in BMI.

**Table 1:** Summary of demographic data from participants who undertook reproducibility assessment of wave near infrared spectroscopy (NIRS), those who undertook all bone health testing, and sub group data of the latter based on sex.

Demographic	Reproducibility only (*N*=41)	All testing (*N*=36)	Males (*n*=22)	Females (*n*=14)	value of *p* between sexes
Age (years)	57.7 (14.2)	62.0 (8.4)	61.8 (9.4)	62.3 (7.0)	0.86
Height (m)	1.71 (0.09)	1.70 (0.09)	1.74 (0.08)	1.63 (0.06)	<0.001
Weight (kg)	77.7 (11.9)	76.8 (11.3)	80.2 (9.5)	71.54 (12.3)	0.02
Body mass index (kg/m^2^)	26.6 (3.2)	26.5 (2.9)	26.3 (2.4)	26.7 (3.6)	0.72
Sex	27m/14f	22m/14f			
Diastolic blood pressure (mmHg)	76 (8.1)	77 (9)	77 (7)	77 (7)	0.92
Systolic blood pressure (mmHg)	132 (17.8)	133 (17)	134 (17)	131 (17)	0.60
Pulse (beats/min)	66 (11.8)	64 (9)	62 (9)	68 (7)	0.05
Resting arterial oxygen saturation (%)	97 (1.6)	97 (1)	97 (1)	98 (2)	0.06
Resting foot temperature (°C)	29.1 (2.0)	28.8 (2.1)	28.7 (2.0)	28.9 (2.2)	0.80
Knee circumference (mm)	380 (25)	379 (26)	378 (20)	382 (33)	0.63
Calf circumference (mm)	373 (25)	372 (26)	373 (22)	369 (31)	0.62
Ankle circumference (mm)	251 (17)	251 (16)	257 (13)	242 (17)	0.01
Right ankle brachial index		1.26 (0.16)	1.26 (0.17)	1.26 (0.14)	0.91
Left ankle brachial index		1.24 (0.13)	1.23 (0.14)	1.24 (0.12)	0.82
Menopausal duration (years)			n/a	14.4 (10.4)	n/a

Results presented are mean values presented with SD in parentheses. values of *p* are from independent *t*-tests between sexes.

### Reproducibility

There was a mean of 12days between testing and retesting of haemodynamic markers in bone and calf muscle derived by NIRS (SD 7days). Tables 2 and 3 summarise the DO and PO reproducibility data of the NIRS haemodynamic markers of the proximal tibia and lateral calf presented in Figure 2. Of the 41 participants tested, usable test/retest data of the proximal tibia were achieved from 26 participants and from 30 participants at the lateral calf. Reasons for missing data were predominately from nTHI changes of >15% during an occlusion; two incidences of optode movement, one instance where HHb did not mirror O_2_Hb changes; one instance of equipment error where nTHI data were not recorded; and, one participant withdrawal due to the discomfort of occlusions. Within the participant data included, some individual haemodynamic markers assessing rates of change were removed when Pearson’s *r*-value was <0.9 and/or where the condition of a <5% change in nTHI within the 60s measurement period was not met, indicating potentially incomplete arterial occlusion.

**Table 2:** Reproducibility data on NIRS markers of the proximal tibia (as presented in Figure 2).

	TOI_rest (%)	TOI_DO_60s (%/s)	TOI_DO_absΔ (%)	HHb_DO_60s (μM.cm/s)	HHb_DO_absΔ (μM.cm)	O_2_Hb_DO_60s (μM.cm/s)	O_2_Hb_DO_absΔ (μM.cm)	TOI_PO_20s (%/s)	TOI_PO_absΔ (%)	O_2_Hb_PO_20s (μM.cm/s)	O_2_Hb_PO_absΔ (μM.cm)
Paired data (N)	26	22	26	26	26	26	26	23	26	26	26
Mean	78.4	−0.026	−7.3	0.326	98.9	−0.329	−99.2	0.256	7.8	7.328	186.2
Between participant SD	4.2	0.005	2.2	0.107	36.1	0.110	55.8	0.117	2.6	2.803	59.5
Within participant RMSSD	2.3	0.004	1.9	0.074	24.7	0.094	23.9	0.100	2.3	1.114	24.8
Within participant RMSCV (%, with 95% CI)	3.0 (0–6.0)	16.3 (0–36.3)	24.8 (0.1–49.4)	25.9 (0–56.1)	31.4 (0–73.1)	29.9 (0–62.1)	27.7 (0–58.6)	43.0 (3.8–82.1)	27.6 (0–57.6)	19.1 (0.0–39.7)	18.6 (0–41.0)
Repeatability coefficient	6.4	0.011	5.2	0.204	68.4	0.261	66.2	0.278	6.4	3.086	68.8
ICC (with 95% CI)	0.71 (0.46–0.86)	0.28 (−0.14–0.62)	0.36 (0.0–0.65)	0.53 (0.19–0.76)	0.55 (0.21–0.77)	0.33 (−0.05–0.63)	0.83 (0.67–0.92)	0.15 (−0.26–0.52)	0.30 (0.0–0.61)	0.86 (0.71–0.93)	0.85 (0.69–0.93)

Markers in grey were used for further comparison with bone health markers. SD, standard deviation; RMS, root mean square; CV, coefficient of variation; ICC, intra class correlation; TOI, total oxygenation index; HHb, deoxygenated haemoglobin; O_2_Hb, oxygenated haemoglobin; DO, during occlusion; and PO, post occlusion.

**Table 3:** Reproducibility data on NIRS markers of the lateral calf (as presented in Figure 2).

	TOI_rest (%)	TOI_DO_60s (%/s)	TOI_DO_absΔ (%)	HHb_DO_60s (μM.cm/s)	HHb_DO_absΔ (μM.cm)	O_2_Hb_DO_60s (μM.cm/s)	O_2_Hb_DO_absΔ (μM.cm)	TOI_PO_20s (%/s)	TOI_PO_absΔ (%)	O_2_Hb_PO_20s (μM.cm/s)	O_2_Hb_PO_absΔ (μM.cm)
Paired data (N)	29	27	29	29	29	28	28	30	29	29	28
Mean	68.8	−0.060	−17.0	0.874	246.7	−0.755	−182.3	1.232	22.9	18.92	354.4
Between participant SD	4.3	0.023	9.6	0.431	104.3	0.364	88.5	0.439	7.7	7.92	133.5
Within participant RMSSD	2.1	0.011	8.5	0.176	47.0	0.176	37.1	0.249	3.1	3.14	51.3
Within participant RMSCV (%, with 95% CI)	3.1 (0–6.9)	17.2 (1.2–33.2)	22.8 (0–55.5)	18.5 (1.8–35.2)	19.9 (0–40.0)	27.6 (0–56.6)	21.3 (0.7–42.0)	29.1 (0–77.3)	13.7 (0–28.4)	17.0 (0.3–33.7)	13.9 (0–28.3)
Repeatability coefficient	5.8	0.032	23.5	0.487	130.2	0.487	102.6	0.689	8.6	8.71	142.2
ICC (with 95% CI)	0.78 (0.58–0.89)	0.74 (0.51–0.87)	0.29 (0.0–0.59)	0.84 (0.69–0.92)	0.81 (0.63–0.90)	0.79 (0.59–0.89)	0.80 (0.62–0.90)	0.78 (0.60–0.89)	0.35 (0.0–0.63)	0.84 (0.69–0.92)	0.84 (0.69–0.92)

SD, standard deviation; RMS, root mean square; CV, coefficient of variation; ICC, intra class correlation; TOI, total oxygenation index; HHb, deoxygenated haemoglobin; O_2_Hb, oxygenated haemoglobin; DO, during occlusion; and O, post occlusion.

Tables 2 and 3 demonstrate comparable reproducibility results for the proximal tibia and lateral calf. The 60s rate of change for TOI during occlusion (TOI_DO_60s) is the best performing DO marker in terms of RMSCV. The 60s rate of change for HHb during occlusion (HHb_DO_60s) was the next best alternative DO marker identified, with the absolute change in O_2_Hb across 4min during occlusion (O_2_Hb_DO_absΔ) the best performing O_2_Hb marker DO for both the proximal tibia and lateral calf.

In terms of post occlusion markers, the 20s rate of recovery post occlusion for O_2_Hb (O_2_Hb_PO_20s), and the absolute maximum change in O_2_Hb and TOI (O_2_Hb_PO_absΔ and TOI_PO_absΔ, respectively) were the best performing markers at the proximal tibia and lateral calf in terms of RMSCV and when comparing the magnitude of RC relative to the typical mean score across the cohort. These PO markers also had strong ICC results of greater than 0.8.

**Figure 3:**
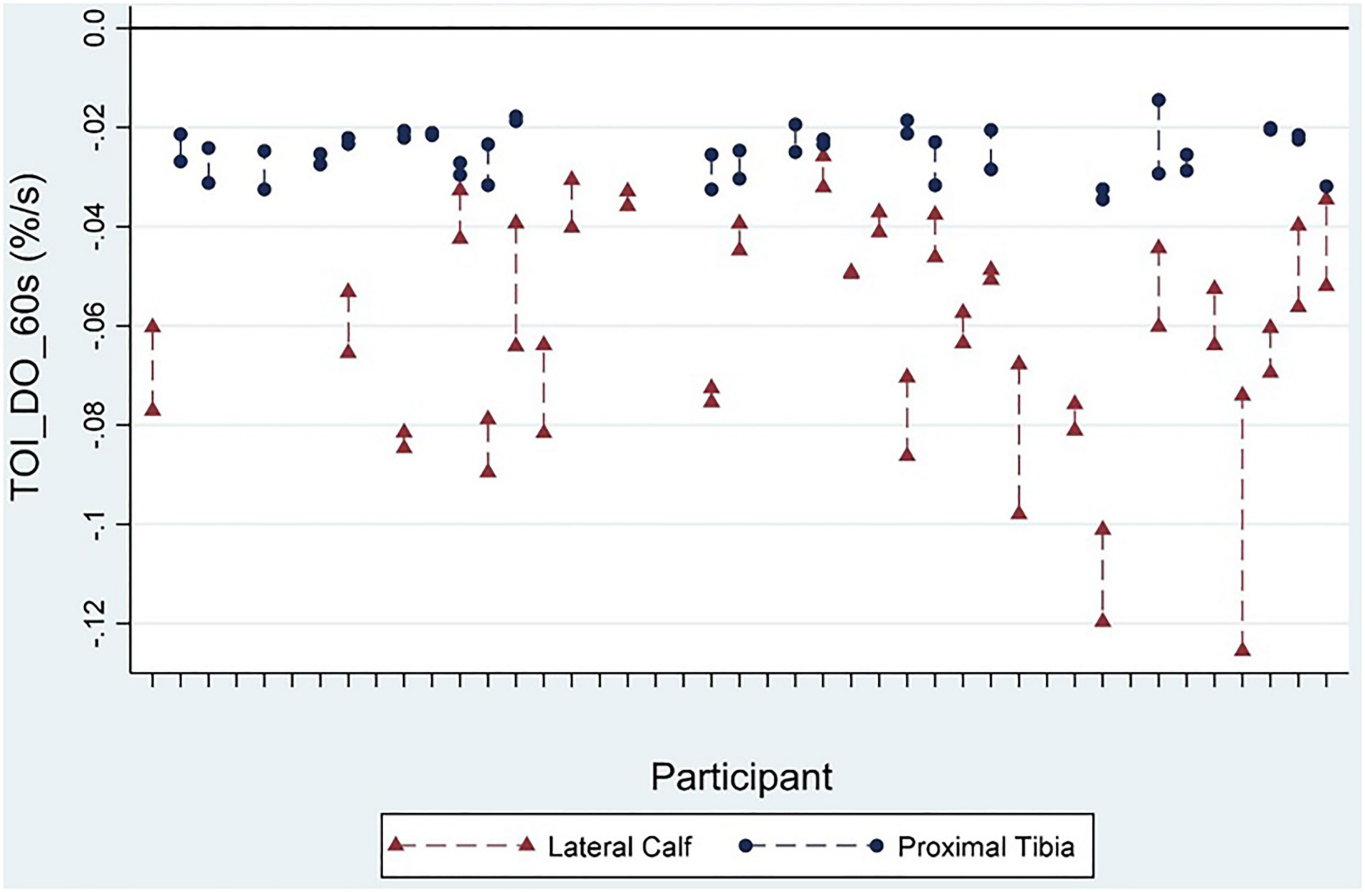
Graphical demonstration of test/retest scores of the TOI_DO_60s marker for the proximal tibia and lateral calf representing the rate of TOI reduction during the last 60s of arterial occlusion. The relatively low between-participant variation at the proximal tibia contributes to a lower ICC score than at the lateral calf [0.28 (95%CI −0.14–0.62) vs. 0.74 (95%CI 0.51–0.87), respectively], despite comparable root mean square coefficient of variation (RMSCV) with the lateral calf [16.3% (95%CI 0–36.3%) vs. 17.2% (95%CI 1.2–33.3%), respectively].

These best performing DO and PO markers were used for further analysis when comparing between the proximal tibia and lateral calf, and investigating associations with NIRS results and other tests of bone health. It was noted that TOI markers typically had lower ICCs compared with corresponding HHb and O_2_Hb markers, despite having similar or even better RMSCV. This is explainable as RMSCV represents the relative error of within participant error to its mean value, whilst ICC calculations incorporate the ratio of between participant variance to within participant variance (Brandmaier et al., 2018). TOI markers generally had much less variance between participants at the tibia. This may be explained as TOI is a ratio of oxygenated haemoglobin to total haemoglobin, and thus is normalising blood volume changes between participant data, unlike HHb and O_2_Hb markers. Figure 3 provides a more detailed example.

### NIRS Results

**Figure 4:**
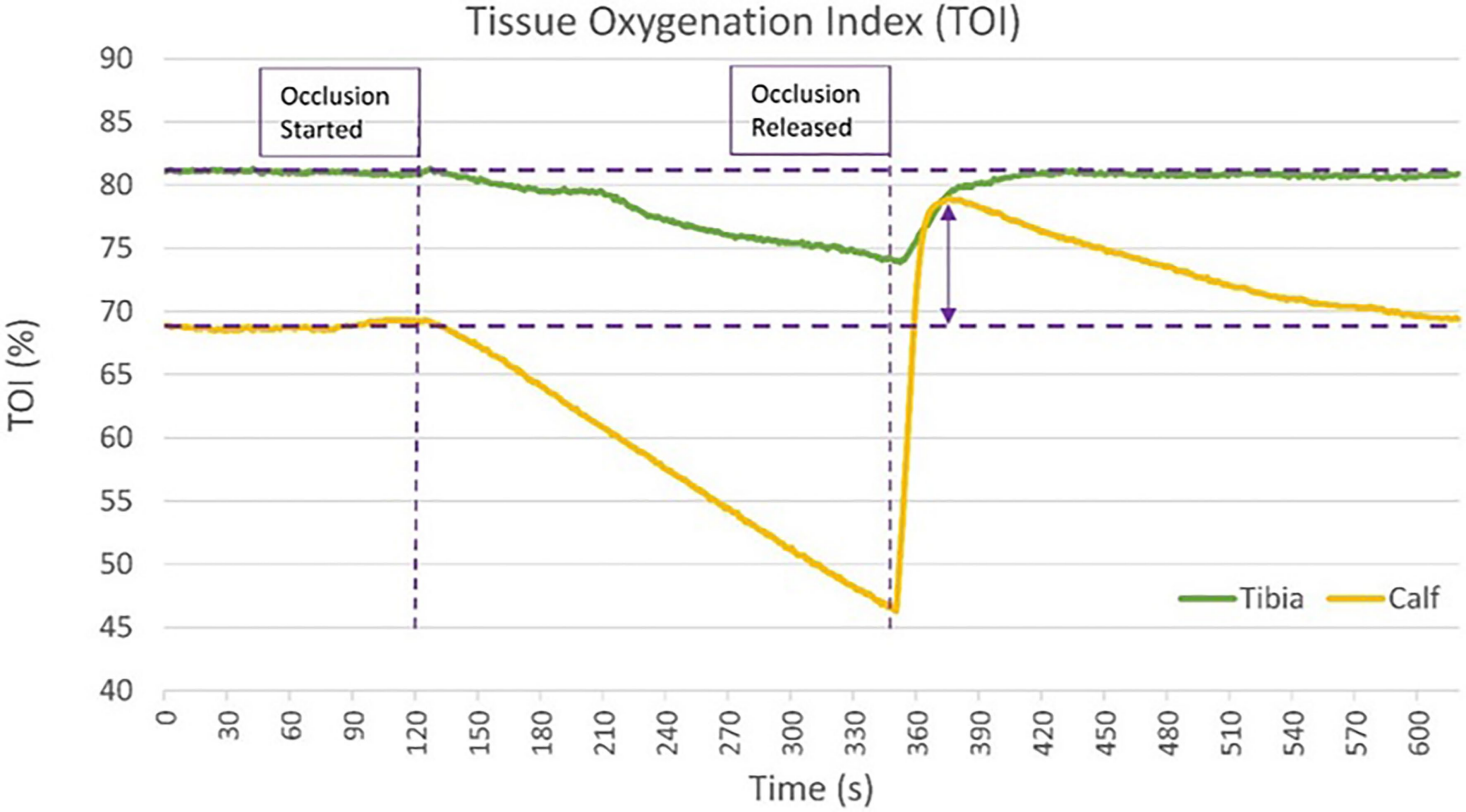
Graphical demonstration of the minimal post occlusive reactive hyperaemic (PORH) response seen at the proximal tibia compared with the lateral calf in one individual participant. Unlike the tibia, the TOI of the calf is seen to “overshoot” the baseline TOI measurement indicated by the purple arrow.

A negligible post occlusive reactive hyperaemic (PORH) response in TOI at the tibia was typically observed, despite strong PORH responses at the lateral calf (see Figure 4).

There was a statistically significant difference between the proximal tibia and lateral calf for all DO and PO NIRS markers considered following intra operator reproducibility assessment (*p*<0.05). The calf demonstrated larger DO and PO responses and a lower resting TOI (*p*<0.05) than the proximal tibia.

**Figure 5:**
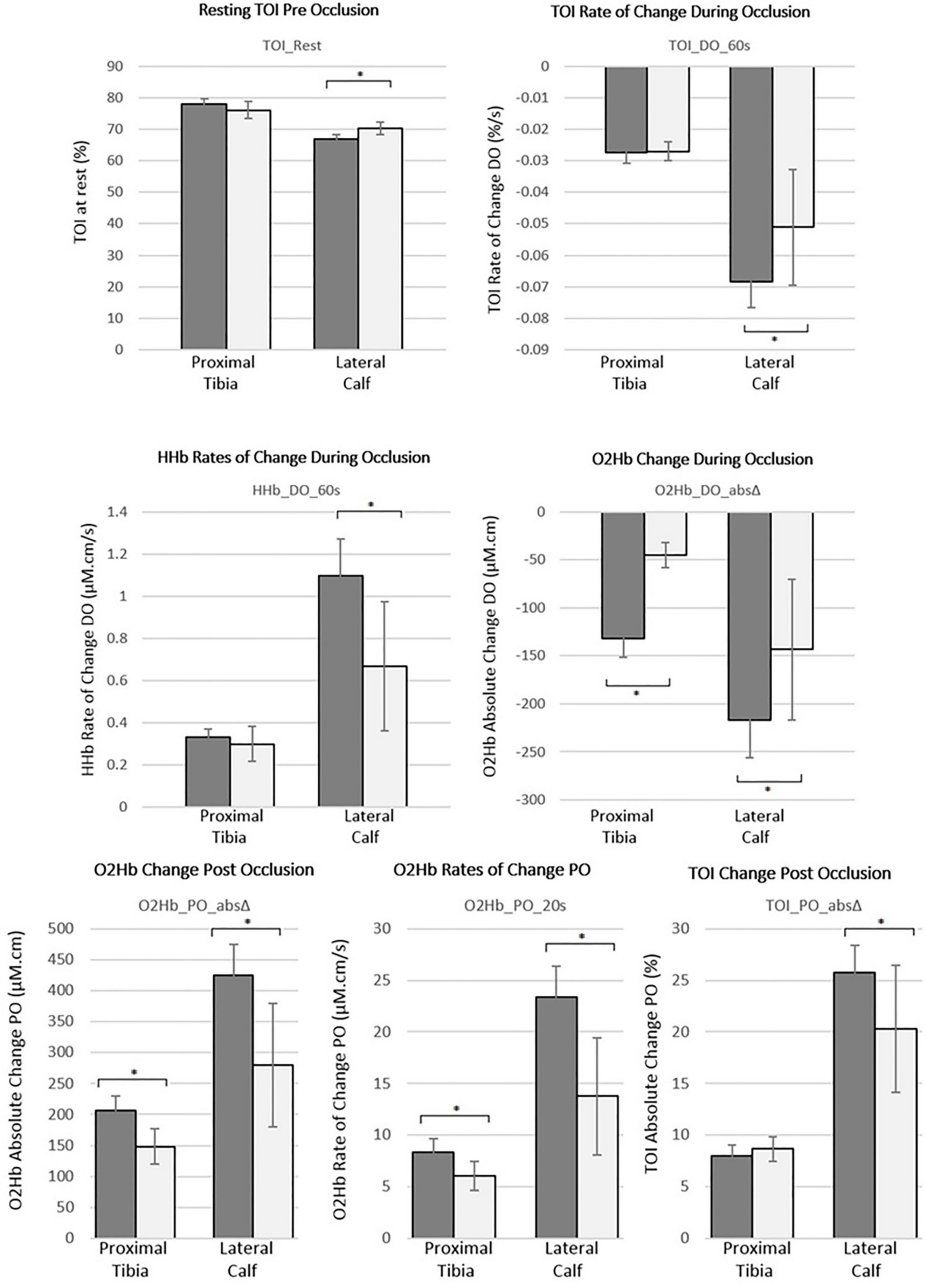
Summary of mean haemodynamic markers for both the proximal tibia and lateral calf stratified by sex [male=dark (*n*=22); female=light (*n*=14)], based on the best performing haemodynamic markers from reproducibility assessment. Error bars represent 95% CIs of mean values and ^*^denotes statistically significant differences (value of *p*<0.05) based on independent *t*-tests between sexes.

Figure 5 demonstrates these markers graphically for both the proximal tibia and lateral calf, stratified by sex. Females had significantly reduced oxygen extraction compared to males during occlusion at the lateral calf as indicated by statistically significant reductions for all three DO markers (*p*<0.05). These were also reduced in females at the proximal tibia but only statistically significant for the O_2_Hb_DO_absΔ marker (*p*<0.001). Post occlusion recovery was also reduced significantly in females compared to males with both O_2_Hb_PO_20s and O_2_Hb_PO_absΔ markers demonstrating statistically significant differences between sexes at both the proximal tibia and the lateral calf (*p*<0.05), whilst TOI_PO_absΔ was only significantly different at the lateral calf (*p*<0.05).

There was evidence that DO and PO responses were associated at the lateral calf, with the absolute change in O_2_Hb concentration during occlusion (O_2_Hb_DO_absΔ) correlating strongly with the absolute change in O_2_Hb concentration post occlusion release (O_2_Hb_PO_absΔ; *r*=−0.91; *p*<0.001). Although weaker, this correlation was also statistically significant at the proximal tibia (*r*=−0.70; *p*<0.001).

As expected, after occlusion release, the rate of O_2_Hb recovery (O_2_Hb_PO_20s) correlated strongly with the absolute O_2_Hb concentration change (O_2_Hb_PO_absΔ) at both the tibia and calf (*r*=0.88; *p*<0.001 and *r*=0.98; *p*<0.001, respectively), suggesting the rate and extent of reoxygenation are linked during recovery from ischaemic events.

### MRI Results

Table 4 presents summary MRI results, including sub groups based on sex. Males had a significantly larger cross sectional area of bone at the proximal tibia, in line with their significantly greater height (as reported in Table 1). MRS results demonstrate a very high fat fraction in all participants with a narrow range of results (from 91.2 to 98.5%), suggesting yellow marrow at the proximal tibia measurement site for all participants. There was little evidence of differences in fat fraction between sexes. Weak non-significant associations were observed between MRS fat fraction and all haemodynamic markers obtained with NIRS, and with DXA measurements, most likely due to the narrow range of fat fraction results observed amongst participants.

**Table 4:** Summary of MRI measurements taken at the proximal tibia at the site of NIRS measurements, including sub groups based on sex (SD in parentheses).

	Total (*N*=35)	Males (*n*=21)	Females (*n*=14)	value of *p* between sexes
Total axial bone area (mm^2^)	939.9 (243.1)	1030.2 (255.2)	804.3 (146.3)	0.005
Cortical axial bone area (mm^2^)	320.7 (59.2)	350.3 (44.6)	280.4 (53.6)	0.001
MRI spectroscopy fat fraction (%)	96.1 (1.8)	96.1 (2.0)	96.1 (1.4)	0.84

Values of *p* are from independent *t*-tests between sexes.

### DXA Results

Table 5 summarises results obtained from DXA scans. Males had higher BMD than females across all sites, with statistically significant higher total body BMD, leg BMD, tibial BMD, and overall percentage bone tissue, in line with expected sex-dependent BMD changes associated with the post-menopausal women recruited in this study (Prisby, 2017). Males also had significantly reduced total body fat percentage and lower leg fat percentage compared with females.

**Table 5:** Summary of mean areal bone mineral densitometry (BMD; in g/cm^2^) and associated dual x-ray absorptiometry results including sub groups based on sex status (SD in parentheses).

	Total (*N*=36)	Males (*n*=22)	Females (*n*=14)	value of *p* between sexes
L1–L4 BMD	1.168 (0.150)	1.189 (0.152)	1.135 (0.146)	0.30
Average hip BMD	1.049 (0.124)	1.068 (0.103)	1.019 (0.151)	0.25
Total body BMD	1.237 (0.096)	1.273 (0.080)	1.179 (0.091)	0.003
Both legs BMD	1.369 (0.142)	1.441 (0.102)	1.256 (0.123)	<0.001
Whole tibia BMD (measured leg)	1.251 (0.144)	1.320 (0.123)	1.144 (0.106)	<0.001
Total body % bone	3.847 (0.447)	4.008 (0.307)	3.594 (0.522)	0.005
Total body % fat	31.56 (7.70)	26.85 (3.74)	38.96 (6.38)	<0.001
Lower leg % fat content	25.2 (9.8)	19.1 (3.9)	34.9 (8.4)	<0.001

Values of *p* are from independent *t*-tests between sexes.

There were a number of statistically significant moderate associations between NIRS markers and BMD at various measurement sites. Table 6 presents a matrix of these associations. Stronger DO and PO responses are considered indicators of better vascular health (Rosenberry et al., 2018), and correlated with increased BMD measurements obtained at several anatomical sites, as well as increased cortical area at the proximal tibia (as measured from axial MRI scans).

**Table 6:** Pearson correlation *r*-values between haemodynamic NIRS markers (graphically demonstrated in Figure 2) taken at the proximal tibia and areal bone mineral density (BMD; in g/cm^2^) at different measurement sites.

	TOI_rest	TOI_DO_60s	HHb_DO_60s	O_2_Hb_DO_absΔ	TOI_PO_absΔ	O_2_Hb_PO_20s	O_2_Hb_PO_absΔ
L1–L4 BMD	0.09	0.17	0.22	−0.24	0.25	0.38	0.45
Total hip BMD	0.03	−0.08	0.39	−0.16	0.00	0.30	0.38
Total body % bone	0.00	0.07	0.46	−0.41	−0.03	0.38	0.39
Total body % fat	−0.12	0.12	−0.34	0.63	0.11	−0.34	−0.42
Total body BMD	0.08	0.02	0.43	−0.51	0.01	0.42	0.56
Both legs BMD	0.16	−0.01	0.44	−0.63	−0.05	0.45	0.57
Whole tibia BMD (measured leg)	0.20	−0.03	0.33	−0.55	0.02	0.37	0.44
Lower leg % fat content	−0.13	0.15	−0.31	0.63	0.08	−0.44	−0.57
Total axial bone area (mm^2^)	0.26	−0.39	0.26	−0.33	−0.16	0.08	0.13
Cortical axial bone area (mm^2^)	0.15	−0.06	0.39	−0.63	−0.08	0.42	0.59

Correlations with percentage tissue composition and cross-sectional area (in mm^2^) are also presented. Statistically significant correlations with value of *p*<0.05 are shaded. TOI, total oxygenation index; HHb, deoxygenated haemoglobin; O_2_Hb, oxygenated haemoglobin; DO, during occlusion; and PO, post occlusion.

Conversely, several NIRS markers show that NIRS DO and PO responses at the tibia were inversely correlated with increasing percentage body fat and lower limb fat. Higher percentage fat content in the lower leg also associated with weaker DO and PO response for all NIRS markers at the lateral calf, with Pearson’s *r*-values ranging in magnitude from 0.61 to 0.79 (*p*<0.001). However, higher fat content in the leg may be confounding for sex-dependent effects as there was also significant differences in percentage fat content in the lower leg between males and females (*p*<0.001, independent *t*-test).

## Discussion

This study shows for the first time that a NIRS arterial occlusion protocol used to assess haemodynamics in bone *in vivo* has high reproducibility and potentially provides useful information about bone health. This includes observed significant differences with the calf muscle, significant sex-based differences, and novel associations with other markers of bone health measured using DXA and MRI. This builds on previous work that has demonstrated NIRS to be reliable and representative of bone during *in vitro* measurements (Takeuchi et al., 1997; Aziz et al., 2010), and earlier *in vivo* feasibility studies in smaller volunteer populations at the proximal tibia of 15 participants or less (Binzoni et al., 2002, 2003, 2006; Klasing and Zange, 2003; Aziz et al., 2010; Alneami, 2015; Siamwala et al., 2015).

### Reproducibility

Our study demonstrated that an AO protocol utilising NIRS can provide reproducible haemodynamic markers of the proximal tibia comparable with values obtained from the lateral calf (Kragelj et al., 2000; Ubbink and Koopman, 2006; Lacroix et al., 2012).

No other studies carrying out a meaningful analysis on the reproducibility of NIRS for measuring bone haemodynamics have been identified. However, the reproducibility results presented in Tables 2 and 3 are in line with alternative applications for measuring haemodynamic markers in bone. For example, Yang et al. (2009) used a pharmacokinetic model to look at dynamic contrast enhanced MRI (DCE-MRI) of bone metastases, with within participant CV ranging between 9 and 15% for markers of contrast agent transfer rates to tissue. Wassberg et al. (2017) reports repeatability coefficients of quantitative standardised uptake markers of 18F-NaF using PET/CT in the range of 23–35% relative to mean results.

Reproducibility results should also be kept in context with the expected minimum clinically important difference of interest for the research application (Buchheit et al., 2011). Griffith et al. (2008) has demonstrated DCE-MRI haemodynamic markers were 50% lower in participants with osteoporosis compared with healthy controls. Libicher et al. (2008) also demonstrated using DCE-MRI areas of Pagetic bone with rates of contrast enhancement more than double that of healthy bone sites. Based on our reproducibility results, meaningful clinical differences of similar magnitude between groups of interest could potentially be identified using NIRS assessment of bone haemodynamics.

Differences in reliability measures between the lateral calf and proximal tibia were observed and may be attributable to the relative heterogeneity of bone tissue leading to a higher reduced scattering coefficient of near infrared light (Elwell, 1995; Jacques, 2013). There is also potentially more error introduced at the tibia due to optode placement, with a smaller target volume compared to the lateral calf.

There were limitations to the assessment of reproducibility. As an intra operator assessment, only one operator and one piece of NIRS equipment has been assessed. Inter operator reliability is expected to be inferior, yet comparable with objective optode positioning and cuff occlusion protocols adopted. The use of multiple systems could be of potential interest in the future, given the previously documented disparities in NIRS results obtained from systems from different manufacturers (Matcher et al., 1995; Yang et al., 2007; Hyttel-Sorensen et al., 2011). There were faster automated occlusion devices available then what was used in this study, and these may improve reproducibility. Non-instantaneous inflation of the cuff may result in some change to total blood volume as the cuff is inflated, as venous flow is momentarily occluded prior to arterial flow (Farzam et al., 2013).

There is a lack of prior consensus on which haemodynamic markers should be utilised from AO protocols. The number of markers considered in our study leaves the statistical analysis open to criticism of multiple testing. However, it was felt this was warranted in order to investigate the most reliable parameters to take forward in this under-researched area. Statistical correction for multiple comparisons was not performed as this was considered inappropriate when comparisons were made between variables that are inherently linked (Bland and Altman, 1995).

### NIRS Observations

Our results give confidence that measurements taken were truly representative of the proximal tibia. Resting TOI rates were significantly different at the proximal tibia when compared to the lateral calf, and in line with existing evidence (Binzoni et al., 2003; Siamwala et al., 2015; Sørensen et al., 2017). The higher resting TOI observed at the tibia compared with the calf may be explained by the lower metabolic rate of bone leading to lower oxygen extraction (Binzoni et al., 2003; Klasing and Zange, 2003). This will result in higher venous oxygen saturation, which has been estimated to contribute up to 70% of the sampled volume in NIRS when measuring muscle (Bakker et al., 2012). As TOI is a ratio of oxygenated haemoglobin to total haemoglobin, relatively smaller microvascular venous reservoirs within bone may also increase TOI (Binzoni et al., 2003).

It was expected the tibia would have a slower resting metabolic rate than muscle (Klasing and Zange, 2003; Wagner et al., 2011), and deoxygenation occurred at a slower rate than at the lateral calf, as has been previously demonstrated (Binzoni et al., 2002, 2003). The magnitude of TOI reduction during AO (mean 7.5%; 95% CI 6.3–8.7%) was comparable with the mean 8.2% reduction (95% CI 7.6–8.8%) in TOI reported at the patella by Farzam et al. (2013). Yu et al. (2005) reports a comparable mean TOI reduction of 16.4% (SD 4.4%) at the calf during occlusion compared with the mean 16.6% TOI reduction observed in our study (SD 5.7%).

The observation of a negligible TOI PORH response at the tibia, despite strong PORH responses at the lateral calf, further strengthens the argument that bone is being sampled by NIRS at the tibia, and that the contribution of overlying soft tissue is minimal, as PORH has been observed in both cutaneous and adipose tissue as well as muscle (Nielsen and Sejrsen, 1972; Thorn and Shore, 2016). It is expected that reduced oxygen extraction rates during occlusion should lead to reduced PORH (Rosenberry et al., 2018), but it is possible with the rigid structure of bone and relatively high intraosseous pressures within bone tissue, that reactive vasodilation of intraosseous microvessels is further limited (Laroche, 2002). Reduced capillary density in bone may also be an explanator for reduced PORH response. Direct comparisons of capillary density between bone and muscle could not be found in the evidence base in either animal or human studies. However, comparison of unrelated murine studies utilising similar immunohistochemistry techniques have reported microvessel density in the plantaris muscle (Waters et al., 2004) and femora (Oikawa et al., 2010), demonstrating a 5-fold greater capillary density in muscle than bone.

Furthermore, large animal studies have shown that vascular resistance can increase in bone marrow during acute exercise-induced ischaemia, with a potential mammalian response of vasoconstriction within bone in order to redirect blood to skeletal muscle during exercise, driven by sympathetic response (Laughlin et al., 2011). This may also potentially explain the lack of PORH response observed in bone although further research is required to confirm this unique regulatory response.

It remains a limitation of NIRS that the underlying physiological reasons for differences between the calf and tibia cannot be definitively elucidated. Differences in AO markers between tibial sites and the lateral calf may be attributable to any combination of differences in local cellular health and oxygen consumption metabolism, blood volume differences, differences in capillary density, and/or differences in vaso-function in response to ischaemia (Nagasawa et al., 2003). Similarly, there is an inherent limitation of the continuous wave NIRS system used that markers are not indicative of absolute blood volume, only haemoglobin concentration changes in response to stimuli.

### Associations With DXA

Our observations of haemodynamic NIRS results associating with BMD provide optimism for potential further use of NIRS in physiological research of bone. Statistically significant correlations were identified between DO and PO markers of NIRS and BMD measurements of the whole body and measured leg. These suggest that stronger AO responses, indicative of superior oxygen extraction DO and superior microvascular regulatory function PO (Rosenberry et al., 2018), associate with higher BMD. Murine studies have shown increased capillary density in both muscle (Waters et al., 2004) and cortical bone (Prisby, 2017) with exercise interventions, with the latter found to precede positive changes to BMD. NIRS could facilitate comparable longitudinal studies in humans.

By demonstrating differences in post-menopausal females with NIRS that are in keeping with other markers of bone health, there is optimism that NIRS may be a useful research tool for observing differences in haemodynamic markers between relevant sub groups. Females demonstrated significantly reduced NIRS markers compared with males DO and PO and had significantly lower BMD at all anatomical sites. It is established that reduced oestrogen levels post menopause can affect both BMD and microvascular function. Prisby et al. (2012) demonstrates a close association between two oestrogen dependent effects post menopause, demonstrating reduced vascular endothelial function and associated reduced trabecular bone volume in an experimental murine study design.

A greater relative fat content in the whole body and the lower limbs of females was observed, despite comparable BMI between sexes. Lifestyle factors around fitness and exercise levels may be confounding here, as this would be expected to affect both vascular NIRS measurements and BMD levels (Lorbergs et al., 2015). Unfortunately, this information was not gathered from participants (Bradbury et al., 2017).

There were no significant associations between TOI markers and BMD at any anatomical site. TOI markers also had low ICC at the proximal tibia, most likely due to relatively small between-participant variance. It is therefore not surprising to see lower correlation with BMD if TOI markers have small variance across the sampled participants. The lack of variation between participants may also be explained by TOI parameters being a ratio value of oxygenated to total haemoglobin, and therefore, normalised for blood volume changes. This may limit TOI parameters at distinguishing response between participants if absolute concentration changes in O_2_Hb and/or HHb are essentially normalised for between-participant comparisons (Suzuki et al., 1999).

The strongest associations were demonstrated involving the two O_2_Hb markers based on absolute concentration change DO and PO. A potential explanation is that the O_2_Hb_DO_absΔ marker is taken over the full 4-min occlusion period, and the O_2_Hb_PO_absΔ marker taken up to maximum hyperaemia. These two markers may better represent these periods during and post induced ischaemia, than the rate markers taken over 60s (DO) and 20s (PO). Previous evidence in muscle and skin has demonstrated differing periods of response during 3–5min occlusions, with initial oxygen extraction in the first 60s occurring faster than the final 60s (Kragelj et al., 2001; Thorn and Shore, 2016). Oxygen extraction rates may also vary as the available oxygenated haemoglobin levels and localised cellular demand of oxygen dynamically change during the occlusion.

### Limitations

Our results have some limitations. Firstly, our findings only represent association between NIRS markers and BMD (as opposed to causation). It is physiologically intuitive that poor microvascular function may precede poor bone density as regulatory bone metabolism is inherently linked with microvascular processes, although, to date there is little direct evidence of this causal pathway *in vivo* in humans, in part due to the difficulties of measuring haemodynamics in bone tissue.

The proximal tibia measurement site was selected as a relatively large, superficial landmark allowing the best chance of reliable proximal AO and subsequent measurement of trabecular bone. The tibia is also a weight bearing bone with precedence in the NIRS evidence base (Binzoni et al., 2002, 2003; Aziz et al., 2010; Siamwala et al., 2015; Sørensen et al., 2017) and likely to be representative of the overall bone health of the participant (Popp et al., 2009). However, MRS results have identified a high fat fraction percentage in all participants suggesting yellow marrow was sampled. Jensen et al. (1990) has previously reported tibial fat fractions of mean 94% (range 92–99%) in 19 normal adult controls, closely replicating results from this study. It has previously been demonstrated that increases in the fat fraction of bone marrow are known to associate with reduced markers of bone perfusion and blood volume (Chen and Shih, 2006). As the range of fat fraction is small in our cohort, it is possible associations between NIRS and other markers of bone health are underestimated, and that a relatively low blood volume in yellow marrow may have reduced reproducibility. Research into adult red marrow sites such as the anatomically superficial sternum are of interest in the future, although, alternative vascular challenges to the AO testing protocol would be required.

An inherent limitation of NIRS measurements is the potential contribution of overlying skin and adipose tissue to the attenuation of NIR light. This has been minimised by recruitment of Caucasian participants with BMI<35kg/m^2^, but this does limit the generalisability of results. Bandages to hold optodes also apply gentle pressure that reduces the thickness of superficial tissue over the proximal tibia and is unlikely to compress microvessels within the dense structure of bone (Mateus and Hargens, 2012; Farzam et al., 2014). The absence of a PORH response in NIRS TOI measurements at the tibia also suggests minimal contribution from overlying soft tissue, as both skin and adipose tissue have been evidenced to show PORH response (Nielsen and Sejrsen, 1972; Thorn and Shore, 2016). Past research has specifically demonstrated minimal contribution of superficial tissue to NIR signal using similar measurement protocols at the tibia (Mateus and Hargens, 2012; Alneami, 2015).

Arterial protocols are known to be potentially painful, which in turn may have varying physiological effects on the microcirculation of participants, especially in response to cardiac and respiratory changes (Gerovasili et al., 2010). This may have contributed to lost data where blood volume changes were greater than 15%. Other plausible explanations for unsuccessful AO are an inadequate application of the cuff or for physiological reasons (for example, calcified arteries not compressing, or deep arteries not affected by the external cuff). Despite some lost data due to participants not tolerating the occlusion, no serious complications were observed resulting from the AO protocols carried out on participants.

### Further Research

If the use of NIRS in bone research is to be further developed, future research should seek to determine how much variation in bone attenuation properties exists between participants due to differences in the BMD and structure of bone. Adjusting for this allows NIRS measurements to solely represent haemodynamic differences between participants. O_2_Hb and HHb measures are derived using MBL algorithms, which assume constant tissue scattering properties, however, this assumption is likely to be violated in comparisons between participants. In our study, DXA observations support variation in bone composition, and there is evidence of variation in cortical thickness at the measurement site. Takeuchi et al. (1997) has demonstrated in an *ex vivo* study that absorption and reduced scattering coefficients of bone are directly associated with BMD using a time resolved spectroscopy system. There is very little evidence around representative absorption and scattering properties of *in vivo* bone in the existing evidence base, and published data varies widely. This may be due to methodological variation (and the difficulties of obtaining these data using existing methods) or a true wide range of attenuation properties of bone across the population (or both; Pifferi et al., 2004; Konugolu Venkata Sekar et al., 2010; Farzam et al., 2013, 2014; Sekar et al., 2016). This issue is not unique to bone tissue, with Van Beekvelt et al. (2017) estimating differences in attenuation properties in muscle may have attributed to up to 12% variation in between-participant results based on the published range of differential path length factors for muscle (ranging between 3.59 and 4.57).

Further research into future technological improvements in NIRS utilising frequency domain and time resolved NIRS systems are recommended and could enhance our understanding of intraosseous haemodynamics. These techniques are able to determine the absorption and scattering properties of sampled bone tissue in real time, providing markers of haemoglobin change adjusted for changes in attenuation properties of the volume sampled, and are also therefore able to produce absolute values of haemoglobin concentration (in μM). O_2_Hb and HHb markers need to be approached tentatively with continuous wave NIRS, as these are taken from an arbitrary baseline. Hence, a change of a certain absolute concentration within a participant could represent any range of relative change when there is an unknown initial absolute haemoglobin concentration value for comparison. Furthermore, with technological advancements of NIRS systems using multiple frequencies there is the possibility of deriving markers of both bone composition and microvascular information in a single device. The use of multi-detector arrays could also better adjust for the contribution of superficial tissue overlying the tibia (Pifferi et al., 2004; Konugolu Venkata Sekar et al., 2010; Sekar et al., 2016; Meertens et al., 2018).

Alongside further research into these improvement of NIRS systems, the results of this study also justify further investment in relevant comparator tests alongside DXA, such as using high resolution peripheral quantitative computed tomography and DCE-MRI at the proximal tibia, which may assist in providing a more complete picture of the specific influences of bone properties which NIRS measurements may represent.

## Conclusion

Results from this study show the potential of several haemodynamic markers derived from NIRS and an arterial occlusion protocol. These reproducible haemodynamic markers are considered to represent the proximal tibia, and the associations with BMD suggest they may prove useful for research applications investigating the intraosseous haemodynamics of bone tissue. This is a significant step forward in providing an *in-vivo* measure of the haemodynamics in bone tissue using non-invasive optical techniques. It is clear further research on the application of NIRS should continue including consideration of the imminent technological advancements in NIR based equipment. If successful, NIRS technology has the potential to become useful when researching common bone diseases like osteoporosis, poor fracture healing, and metabolic bone diseases.

## Data Availability Statement

The raw data supporting the conclusions of this article will be made available by the authors, without undue reservation.

## Ethics Statement

The studies involving human participants were reviewed and approved by Health Research Authority (United Kingdom). The patients/participants provided their written informed consent to participate in this study.

## Author Contributions

RM, KK, WS, and FC contributed to the conception and design of the study. RM and JF collected the data. RM organised the data and performed the analyses with guidance from SB. All authors contributed to the article and approved the submitted version.

## Funding

RM received funding and support from the College of Radiographers Doctoral Fellowship Award (Application 003). SB was supported by the National Institute for Health Research (NIHR) Applied Research Collaboration South West Peninsula. The views expressed in this publication are those of the author(s) and not necessarily those of these supporting institutions.

## Conflict of Interest

The authors declare that the research was conducted in the absence of any commercial or financial relationships that could be construed as a potential conflict of interest.

## Publisher’s Note

All claims expressed in this article are solely those of the authors and do not necessarily represent those of their affiliated organizations, or those of the publisher, the editors and the reviewers. Any product that may be evaluated in this article, or claim that may be made by its manufacturer, is not guaranteed or endorsed by the publisher.
